# The Maternal–Fetal Gut Microbiota Axis: Physiological Changes, Dietary Influence, and Modulation Possibilities

**DOI:** 10.3390/life12030424

**Published:** 2022-03-15

**Authors:** Eva Miko, Andras Csaszar, Jozsef Bodis, Kalman Kovacs

**Affiliations:** 1 Department of Medical Microbiology and Immunology, Medical School, University of Pécs, 12 Szigeti Street, 7624 Pécs, Hungary; 2 National Laboratory for Human Reproduction, University of Pécs, 7624 Pécs, Hungary; csaszar.andras@pte.hu (A.C.); bodis.jozsef@pte.hu (J.B.); kovacs.kalman@pte.hu (K.K.); 3 Janos Szentagothai Research Centre, 20 Ifjusag Street, 7624 Pécs, Hungary; 4 Department of Obstetrics and Gynaecology, Medical School, University of Pécs, 17 Edesanyak Street, 7624 Pécs, Hungary

**Keywords:** gut microbiota, pregnancy, fetal development, maternal nutrition, probiotics

## Abstract

The prenatal period and the first years of life have a significant impact on the health issues and life quality of an individual. The appropriate development of the immune system and the central nervous system are thought to be major critical determining events. In parallel to these, establishing an early intestinal microbiota community is another important factor for future well-being interfering with prenatal and postnatal developmental processes. This review aims at summarizing the main characteristics of maternal gut microbiota and its possible transmission to the offspring, thereby affecting fetal and/or neonatal development and health. Since maternal dietary factors are potential modulators of the maternal–fetal microbiota axis, we will outline current knowledge on the impact of certain diets, nutritional factors, and nutritional modulators during pregnancy on offspring’s microbiota and health.

## 1. Introduction

**Figure 1:**
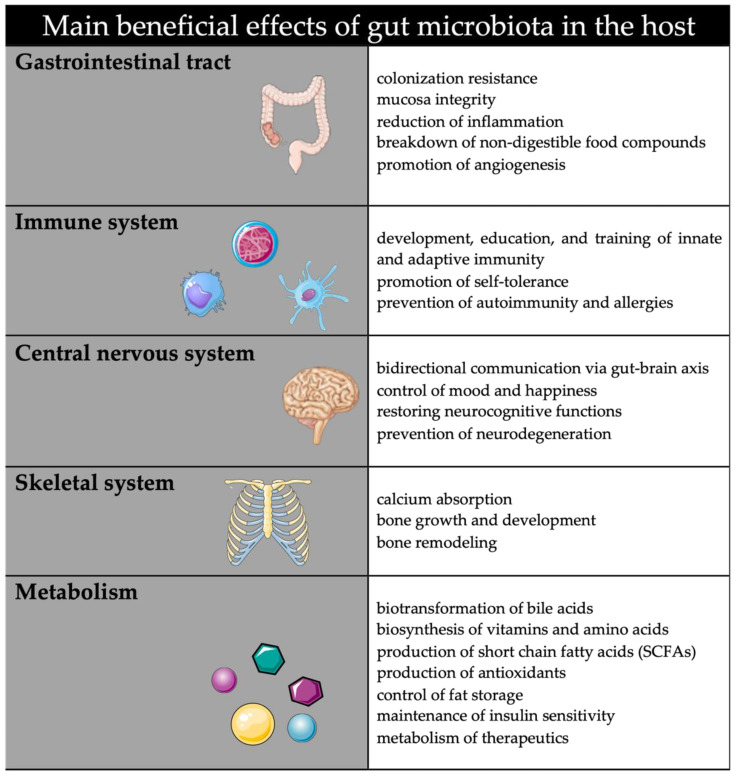
Suggested functional effects of gut microbiota in host organism.

The term “microbiota” defines the entirety of microorganisms that reside in the organs and tissues of an individual acting mostly commensal or symbiotic [1]. The microbiota includes bacteria, archaea, fungi, and viruses, from which bacterial microbiota is the best characterized and most intensively studied component. While the human body hosts many trillions of bacteria, the gastrointestinal tract is the most densely colonized area, with bacterial concentrations ranging from 10^1^–10^3^ bacteria/gram tissue in the upper intestine to 10^11^–10^12^ bacteria/gram tissue in the colon [2,3]. Analyzing the ratio of intestinal bacterial phyla, *Firmicutes* (species, e.g., *Clostridiales, Lactobacillus, Enterococcus*) and *Bacteroidetes* (species, e.g., *Bacteroides*) make up the majority, with less dominance of the other phyla *Actinobacteria (Bifidobacteria), Proteobacteria (Escherichia coli), Fusobacteria*, and *Verrucomicrobiota* [4,5,6]. In recent years, the human microbiota, especially the intestinal microbiota, has been recognized as having a major impact on human health, contributing to different physiological processes. The intestinal bacterial community is thought to participate in the metabolic, biochemical, and immunological balance of the host organism (summarized in Figure 1) [7,8,9,10].

Since the development of the human gut microbiota begins probably already before birth, it can be assumed that maternal and gestational factors and environmental exposures during pregnancy could affect healthy development and composition of fetal/neonatal/infant gut microbiota and thereby offspring’s health issues [11,12,13].

The focus of this review, therefore, is to summarize the main characteristics of maternal gut microbiota and its possible consequences on fetal development and offspring’s health. Besides genetic and environmental factors, nutrition is a key determinant factor affecting the composition and function of gut microbiota; therefore, we will discuss the effects of maternal dietary factors and modulation possibilities on pregnancy microbiota [14,15].

## 2. Maternal Gut Microbiota

Pregnancy represents a challenging condition for the maternal organism. To meet fetal requirements and thereby ensure self-integrity, it must undergo several profound physiological changes. Maternal adaptation involves primarily endocrine, metabolic, and immunological changes. During pregnancy, the notable rise of progesterone, estrogen, and thyroid hormone levels is well known. Metabolic alterations focus on the expanding neonatal nutrient and energy demand: food intake, insulin secretion, and lipogenesis will increase significantly, resulting in a metabolic syndrome-like condition [16]. Immunological changes are referred to as maternal–fetal immune tolerance: very special and tight regulation of tolerogenic and proinflammatory immune responses. These immune mechanisms enable successful implantation, along with sufficient placentation on the one hand and restoration of maternal antimicrobial immunity on the other [17,18]. In the periphery, healthy human pregnancy is characterized by a mild systemic inflammatory response [19,20].

In the last decade, it has become obvious that pregnancy affects the composition of the maternal gut microbiota, indicating another major pregnancy-related maternal change with possible consequences for fetal/neonatal development. While in the first trimester of pregnancy, the intestinal microbiota is comparable to that of healthy, nonpregnant women, the composition of the gut microbial community changes significantly from the first to the third trimester [21,22]. From the second trimester onwards, *Proteobacteria*, *Bifidobacteria*, and lactic-acid-producing bacteria (some specific *Lactobacillus* strains) increase parallel with the reduction of the number of butyrate-producing bacteria [7]. Overall, the gestational gut microbiota is characterized by a low alpha diversity index (intraindividual bacterial diversity) and a high beta diversity index (interindividual bacterial diversity), with the most prominent changes occurring mainly in the third trimester [11,23,24,25]. Intestinal microbiota transfer from pregnant women to germ-free mice revealed functional consequences of changes in gut microbiota during pregnancy [22]. Microbiota-transplanted mice gained weight and showed impaired glucose tolerance associated with insulin resistance [22]. These data suggest that the described changes in the intestinal microbiota during pregnancy might contribute to the well-known metabolic changes observed in pregnant women. Moreover, an increased *Proteobacteria* ratio is thought to stimulate the immune system, leading to enhanced local inflammatory responses. Inflammation, in turn, increases gut mucosa permeability and enables bacterial translocation [22]. This, at least in part, provides a possible reason for the mild systemic inflammation observed in the peripheral blood of healthy pregnant women [19,20]. It should be mentioned that changes in maternal microbiota composition could be influenced by many patient-related factors (maternal diet, maternal BMI before conception, weight gain during pregnancy, and metabolic diseases) and also by population level (ethnicity, geographic, and environmental factors) [7,12,13,14,19,25,26,27,28,29,30,31,32,33,34,35].

## 3. Establishment of the Maternal–Fetal Gut Microbiota Axis

**Figure 2:**
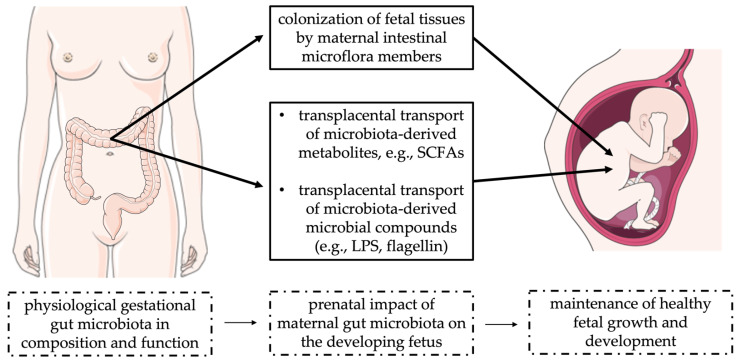
Possible action of maternal gut microbiota on the developing embryo during pregnancy.

The impact of maternal gut microbiota on fetal growth and development represents a major field of investigations and theories. Two main distinct pathways were proposed on how gestational intestinal microbiota could exert significant effects on the fetal side (summarized in Figure 2).

### 3.1. Placental Microbiota

One of the theories suggests direct and beneficial effects of bacterial presence assuming colonization of fetal tissues by maternal microbes in utero, long before birth [11]. Prenatal microbial transport from the maternal gastrointestinal tract to the fetus is only a hypothetical consideration requiring direct evidence in the future. According to actual presumptions, microbes at the maternal site are translocated somehow from the intestinal epithelium into the bloodstream and then delivered to the placenta. The bacterial transition could be facilitated through alteration of maternal gut microbiota composition during pregnancy. The increasing abundance of the phylum *Proteobacteria* during pregnancy is thought to be associated with proinflammatory changes (increased serum endotoxin and zonulin concentrations, as well as increased fecal calprotectin levels) in the bacterial environment, enhancing mucosal permeability and microbiota translocation (cellular uptake and occurrence of intestinal microbiota members in extraintestinal tissues and maternal circulation probably resulting in colonization of fetal gut in utero) [36,37].

Regarding the origin of neonatal gut microbiota, there was a consensus about its postnatal establishment until the last two decades. The uteroplacental unit was considered as being sterile. Bacterial occurrence was thought to be associated with colonization and subsequent infection mostly through the ascending way, leading to chorioamnionitis in most cases. The dogma of the “sterile womb” was widely accepted with the consideration that this sterile environment would protect the fetus from infections [13,38,39].

Over the last two decades, noncultivational, PCR, and DNA sequencing-based data have emerged, supporting new theories about maternal–fetal transmission of microbes in utero [13,40,41,42,43,44,45,46,47,48]. Convincing animal studies have further supported this route of transmission. Increased bacterial translocation from the gut to extraintestinal tissues was observed in pregnant and lactating mice [49]. Orally administered, foreign bacteriophage DNA to mice was shown to persist in the gastrointestinal tract to penetrate the intestinal epithelium and could be discovered in fetuses and newborn animals through the transplacental pathway [50,51]. Moreover, orally inoculated pregnant mice with genetically labeled *E. faecium* strain transmitted labeled bacteria to the amniotic fluid and to the fetal gut during pregnancy [52,53]. Interestingly, the murine fetus seems to be exposed to viable and cultivable bacteria in midgestation and to noncultivable bacteria in late gestation [47].

The human placental microbial community was found to be dominated by the major phylum *Proteobacteria*. The composition was comparable to the oral microbiota, with the species of *Prevotella* and *Neisseria* suggesting the hematogenic route of seeding from the oral cavity to the placenta [46]. It is of note that during pregnancy, the viable oral microbiota increases in number with the parallel rise of the parodontopathogenic strains *Porphyromonas gingivalis* and *Aggregatibacter actinomycetemcomitans* in the subgingival plaque [54,55]. In pregnant mice, oral infections with *Campylobacter rectus* and *Porphyromonas gingivalis* or *Fusobacterium nucleatum* resulted in inflammatory placental and fetal complications [56,57,58]. In humans, pregnant women diagnosed with periodontal disease showed an increased risk of pregnancy complications. This observation could be interpreted as the result of an enhanced bacterial transition from the inflamed oral mucosa with increased permeability to the uteroplacental unit [59,60,61,62].

Microbiota studies were not only limited to the investigation of the placenta but reported physiological bacterial presence in the amniotic fluid, in the umbilical cord, fetal gut, and also in the meconium [13,21,39,43,63,64,65]. So far, all of them were considered sterile before.

With the increasing number of conducted studies suggesting in utero fetal exposure to maternal microbiota, concerns have been raised regarding the interpretation of their obtained results [66]. Possible contamination of samples with low microbiota density is a major issue with data distortion potential. Avoiding contamination is very challenging since multiple sources exist. For example, laboratory reagents (nucleic acid extraction kits and PCR reagents) can harbor low concentrations of bacterial DNA, and samples may be contaminated at the time of or even before collection [67,68,69]. Using the most appropriate analysis platform is also another important issue [70]. Whether in utero exposure of fetal tissues to maternal microbiota members exists is still controversial, and there is disagreement even among the experts in the field.

Another argument against the in utero colonization hypothesis is the fact that germ-free animals are usually generated from non-germ-free pregnancies by embryo transfer following hysterectomy. This could not be the case if mammalian fetuses were already contacted by maternal microbiota members during pregnancy [39].

Usually, the intestinal epithelial barrier itself acts as a physiological barrier, even for the entry of members of the harmless microbiota. Dendritic cells (DCs) can take up bacteria intracellularly from the gut lumen and transport them first to the lymph nodes locally. From here, the bacterial spread can also continue widely, e.g., into the bloodstream, resulting finally in transplacental trafficking [71,72,73]. This concept of hematogenic maternal intestinal microbiota translocation to the fetus was strengthened by mouse experiments, where increased bacterial sequestering in murine mesenteric lymph nodes was demonstrated [49].

Once bacteria arrive at the fetal site, they probably get noticed. One of the most possible ways of recognizing foreign structures is through toll-like receptors (TLRs). Human TLRs represent a family of 10 transmembrane proteins. They are located either on the cell surface or in intracellular vesicles of primary sentinel cells of innate immunity (macrophages, dendritic cells, and mast cells) in most human tissues [74]. TLRs function as conserved innate immune receptors, recognizing pathogen-associated molecular patterns (PAMPs) that are broadly shared by microbes but not by the host itself. There are different types of TLRs for the recognition of distinct bacterial structures resulting in classical immune activation and inflammatory response directed against the pathogen [75,76,77].

Despite comparable expression levels of TLRs on neonatal monocytes, the extent of activation of the TLR pathway is considerably reduced compared to adults [77]. Reduced levels of proinflammatory cytokines produced by neonatal monocytes, limited expression of TLR-associated intracellular signaling proteins, and impaired phosphorylation activity of TLR-induced protein kinases suggest immature innate immunity and ongoing immune development in the perinatal period [78,79]. Within this immune milieu, fetal recognition of maternally derived microbiota members exposed in utero would likely result in inadequate immune response favoring immune tolerance of the translocated bacteria. Supporting this concept, recent studies revealed the presence of effector memory T cells in second-trimester fetal tissues [68,80]. However, it should be mentioned again that the concept of existing prenatal microbiota before birth is a matter of debate, and it is not widely accepted. Further studies are needed in the future to clarify the possibility of maternal–fetal microbiota exchange.

### 3.2. Effects of Microbiota-Derived Molecules

The second possible pathway of regulating fetal growth and development through the maternal intestinal microbiota is indirect. It is thought to be mediated by microbiota-derived metabolites that are transmitted transplacentally to the fetus [81,82,83,84]. These soluble factors are either synthesized endogenously by members of the microbiota or are metabolites of compounds that are taken up from the intestinal lumen.

One of the most convincing proofs of this concept comes from animal studies. Reversible colonization of germ-free murine pregnant females with a nonpathogenic *E. coli* strain resulted in changes in the intestinal innate immune system development of the offspring [85,86]. Proliferation of innate lymphoid cells type 3 (ILC3s), an innate cell population critical for intestinal barrier functions and host defense, was observed, suggesting maternal microbiota-derived aryl hydrocarbon receptor (AhR) ligands [87]. Moreover, maternal colonization alters the gene expression profile of the offspring’s gut epithelium. Expression of genes encoding homeostasis, integrity, and differentiation (upregulated gene networks for cell division and differentiation, mucus and ion channels, metabolism of xenobiotics, bile acids, complex lipids, and sugars) was modulated in small intestinal epithelial cells of offspring born to mothers who had experienced reversible colonization during pregnancy [86].

SCFAs are considered to be the main soluble end product of bacterial metabolism, with a major impact on an individual’s health issues. They are taken up by the gut epithelium and transported to the tissues via circulation [88]. During pregnancy, SCFA concentrations (e.g., levels of acetic acid, propionic acid, butyric and caproic acid) in the cecum increase significantly [89]. The dominant SCFA in both pregnant women and their babies is acetic acid [90]. SCFAs act as signaling molecules through G-protein receptors (GPR), mainly through GPR41 and GPR43 [88]. SCFAs from the maternal gut microbiota can be sensed through uteroplacental GPR41 and GPR43 receptors [91,92]. A series of murine studies demonstrated the beneficial effects of SCFAs during embryo development [91,92,93,94]. SCFAs are responsible for increasing free fatty acids’ oxidation and mitochondrial activity in muscle and brown adipose tissue [95]. Their beneficial effect on metabolism, mainly through the control of insulin levels, was also observed in the fetus [93]. SCFAs have a major impact on the developing immune system, especially on immune regulatory mechanisms. They may control and balance immune responses, thereby preventing exaggeratory inflammation but also autoimmunity. Regulatory T-cell (Treg) proliferation, differentiation, cytokine synthesis, Foxp3 expression, and anti-inflammatory activities were found to be promoted by SCFAs [88,94,96].

Another suggested major function of SCFAs in the fetus is the influence of the development of the nervous system through GPR41 signaling [93]. Enhanced maternal gut microbiota occurs at the third trimester of pregnancy, and this is also a critical period for brain development, such as synaptogenesis, myelination, and development of some specific areas [97,98,99]. Increased microbiota-derived metabolites, such as SCFAs, could have a beneficial effect on neuronal development [99].

The integrity of the intestinal barrier can also be regulated by SCFAs, mainly through the transcriptional regulation of tight junction-related proteins [100].

Although there is no scientific consensus about whether the developing fetus and the placenta are sterile, besides alive bacteria, many endogenous microbial compounds (e.g., lipopolysaccharide (LPS) or flagellin) can reach fetal tissues and get recognized by innate pattern recognition receptors, such as TLRs, mentioned above. Murine experiments revealed continuous penetration of different tissues by bacterial structural elements required for host immune system maturation. In mice, activated T cells can be detected already in the fetal gut, activation is supposed to be the result of antigen recognition from the maternal gut microbiota ([101,102,103,104,105]. Therefore, it can be hypothesized that even without bacterial trafficking, maternal gut microbiota compounds can reach fetal compartments and provoke recognition. The primitive immune system requires interaction with bacteria or at least bacterium-derived molecules in order to learn to distinguish the microbiota from pathogen types in the future [106,107,108,109].

## 4. Effects of Maternal Nutritional Factors on Gut Microbiota and Offspring’s Health

According to epidemiological, clinical, and basic science studies, the offspring’s later health issues can be linked, at least partly, to adverse preconceptional, gestational. and postnatal factors, mainly of maternal origin [110]. Regarding the gut microbiota composition and function, dietary factors could have the most determining potential (Table 1) [111].

### 4.1. High-Fat Diet and Maternal Obesity

Maternal diet type, weight, and nutritional status have an important effect on the developing embryo [112]. Maternal influence on the child’s well-being could be exerted via the intestinal microbiota during pregnancy. This is thought to be regulated at least partly by nutritional factors [113]. The typical Western diet consists of excessive processed foods, dietary fat, and sugars. Such a diet promotes excess weight gain and a dysbiotic gut and is associated with adverse maternal and child health outcomes [113,114,115]. There are numerous fetal developmental characteristics associated with maternal obesity: fetal overgrowth, macrosomia, congenital defects, stillbirth, decreased neonatal Apgar score, preterm delivery, child morbidity, respiratory complications, and neonatal mortality [116,117,118,119,120].

It is well known that the intestinal microbiota is altered profoundly in obese individuals. First, there is an increased abundance of the phylum *Firmicutes* over *Bacteroidetes*, with a reduced microbial diversity [121,122]. Similar findings were observed in rats when diet-induced obesity modulated gut microbiota composition with a lower relative abundance of fecal *Bifidobacterium* spp. and higher relative abundance of *Clostridium* Clusters XI and I [123]. Moreover, murine experiments revealed that the obese phenotype can be transferred to lean germ-free mice via fecal microbiota transplantation [122]. Pregnancy itself further alters the gut microbiota. Reduced numbers of *Bifidobacterium* and *Bacteroides* and increased numbers of *Staphylococcus*, *Enterobacteriaceae*, and *Escherichia coli* were detected in overweight compared with normal-weight pregnant women [28,35,120,122]. These changes in microbiota are thought to be associated with a reduction in butyrate production, a reduction in hydrogen and methane production, and an increase in mucus degradation and local inflammation [124]. Maternal adherence to the dietary reference intake of fat and fiber during pregnancy is thought to be associated with beneficial gut microbiota composition changes, such as higher gut microbiota richness [125]. Maternal microbiota alterations may be transferred to the infant already in utero and during birth. Infants born to obese mothers display a different bacterial microbiota pattern than those born to lean mothers. These differences last at least one year, showing the long-term impact of maternal obesity on offspring’s intestinal microbiota [30,41,120,126]. Similar findings were observed in a primate model, where a high-fat maternal diet (consisting of 36% fat from lard, butter, animal fat, and safflower oil) modulated the offspring’s intestinal microbiome in Japanese macaques [127]. The main changes in humans are differences in the abundances of *Bacteroides* spp., *Enterococcus* spp., *Acinetobacter* spp., *Pseudomonas* spp., *Blautia* spp., *Eubacterium* spp., *Oscillibacter* spp., and *Faecalibacterium* spp. [43,120,126,128,129,130]. There are suggestions that a higher abundance of *Lactobacillus* spp. and a lower abundance of *Bacteroides* spp. The early infant gut microbiota may predict the risk of obesity and overweight in childhood [131]. All these findings support the concept of a vicious intergenerational circle of transferring microbiota patterns related to excessive weight gain and associated unfavorable metabolic development [102].

#### Gestational Diabetes Mellitus: A Special Case

Gestational diabetes mellitus (GDM) is a disease of abnormal glucose tolerance resulting from insulin resistance and showing its first occurrence during pregnancy. Diagnosis of the disease primarily based on the oral glucose tolerance test (OGTT) carried out between 24 and 28 weeks is the gold standard [132].

Obesity and gestational GDM share similar metabolic disorder phenotypes. One of the main suggested mechanisms that could explain insulin resistance and the development of GDM in pregnancy is an unhealthy diet with high fat, high sugar, and low fiber intake characteristics [88,133,134,135]

GDM contributes to changes in the composition of intestinal microorganisms, their diversity, and disturbed SCFA proportions. Distinct microbiota changes can be observed in each trimester. There are some investigations focusing on the dynamic changes of maternal gut microbiota during pregnancy and progression to GDM [136]. The microbiota profile during pregnancy could be a biomarker for early detection of GDM and predict progression of the disease [136,137]. A positive correlation was found between the *Ruminococcaceae* family and glucose level, with a higher odds ratio for diagnosis of GDM [137]. The main findings regarding microbiota changes were: increased relative abundance of the families *Ruminococcaceae*, *Lachnospiraceae*, and *Enterococcaecea*; enrichment of *Bacteroides*, *Blautia*, *Collinsella*, and *Eggerthella* bacteria; decrease in the levels of *Faecalibacterium*; and decrease in alpha diversity in the GDM groups compared to healthy pregnancy [136]. The functional capacity of the GDM gut microbiota revealed an association with enhancement of membrane transport of sugars, oxidative stress responses, branched-chain amino acid transport, and decreased butyrate biosynthesis [136,138]. A possible influence of GDM on fetal/neonatal microbiota was also studied. The placental microbiota from women with GDM harbor lower levels of *Pseudomonadales* order and *Acinetobacter* genus. Moreover, decreased placental *Acinetobacter* was associated with a more adverse metabolic and inflammatory phenotype [44]. The meconium microbiota of offspring of women with GDM showed lower alpha diversity and increased *E. coli* and *Lactobacillus* abundance [139]. Meconium microbiota of infants born to mothers with diabetes is enriched for the same bacterial taxa as those reported in the intestinal microbiota of adult patients [140]. Analyzing microbiota from different body sites immediately after birth varied by the same trend between the maternal and neonatal microbiota, suggesting the intergenerational concordance of microbial variations observed in GDM [140].

### 4.2. Vegetarian Diet

Plant-based and vegetarian eating patterns are very popular nowadays. This diet type is thought to lower the risk for obesity, cardiovascular disease, cerebrovascular disease, diabetes mellitus, and chronic kidney disease [141]. Vegetarian dietary patterns are thought to alter gut microbiota, with beneficial changes for the host (increased SCFAs synthesis, higher abundance of *Bifidobacteria*, *Lactobacilli*, *Roseburia*, *Ruminococcus*, decrease in *Proteobacteria* and *Firmicutes* for instance) [142,143]. Limited information is available regarding gut microbiota of vegetarian pregnant women. One study found no difference in alpha diversity but reduced beta diversity of intestinal microbiota in pregnant vegetarians compared to omnivorous pregnant women [144]. There were also differences in the relative frequency of several genera in those on a vegetarian diet (decrease in *Collinsella* and *Holdemania* and increases in *Roseburia* and *Lachnospiraceae*) [144]. These changes could result in higher SCFA levels associated with healthier gut mucosa and a lower degree of inflammation. No data exist about the impact of gut microbiota of pregnant vegetarian women on the health issues of their offspring [144].

### 4.3. Artificial Sweeteners

In the United States, non-nutritive sweeteners (e.g., sucralose, aspartame, acesulfame-K) are very popular in the daily diet. This is probably due to the growing awareness of sugar’s negative impact on health effects [33,145]. Although several artificial sweeteners almost do not contact the colonic microbiota itself, they seem to change the composition of the gut bacterial community [33]. Based mainly on animal experiments, artificial sweeteners have been shown to alter gut microbiota composition, affecting certain bacterial taxa of adults, as well as their offspring (increase in *Bacteroides*, *Lactobacillus*, and *Clostridiales*, depletion of *Akkermansia muciniphila*). Furthermore, they increase body weight in parallel with activation of energy metabolism bacterial genes involved in carbohydrate absorption, glycolysis, and sugar transport [146,147,148,149,150,151]. Bacterial proinflammatory mediator genes were also shown to increase [147]. *Akkermansia muciniphila*, found depleted in the microbiota after sweetener administration, is a useful bacterium associated with normal weight, balanced serum glucose levels, and intestinal anti-inflammatory effects [33,152,153,154]. Meanwhile, human results also support the concept of adverse effects of artificial sweeteners on offspring’s gut microbiota. Regular intake of artificial sweeteners of women resulted in a higher BMI of one-year-old infants, suggesting that an altered infant gut microbiota could partly account for it since differences of some microbiota-associated metabolites could favor weight gain [155].

### 4.4. Alcohol

Alcohol consumption during pregnancy and its possible consequences on the establishment of intestinal microbiota is, besides artificial sweeteners, another less studied factor. It is well known that regular gestational alcohol use during pregnancy has also been associated with various disorders in neonates [156,157]. Chronic and significant alcohol consumption affects gastrointestinal mucosal integrity and consecutively gut microbiota composition [158,159,160]. It was shown to be associated with decreased intestinal microbiota members such as *Roseburia*, *Faecalibacterium*, *Blautia*, *Bacteroides*, and *Lachnospiraceae*, low levels of butyrate-producing *Clostridiales*, *Bifidobacterium*, and *Lactobacillus*, increased gut permeability, and inflammation [161,162,163,164]. This different microbiota pattern results in enhanced alcohol metabolism and local inflammation [161]. In a study with pregnant mice, reduced *Bacillus* bacteria were observed after ethanol exposure [165]. Little is known about the impact of gestational alcohol consumption on pre- and neonatal microbiota. In one human study, in newborns with mothers admitting alcohol use during pregnancy, an increased ratio in the *Megamonas* genus was observed in the gut microbiota [166]. Interestingly, *Megamonas* was shown to be associated with major depressive disorders, and it is well known that maternal alcohol use affects newborns’ cognitive and behavioral development such as depression and autism [167,168,169].

**Table 1:** Effects of maternal dietary factors on offspring’s microbiota composition.

Author, Year	Study Population	Investigated Fetal Side Microbiota	Method	Main Findings
Maternal High-Fat diet, Obesity				
Collado et al., 2010 [30]	Infants of obese mothers (n = 16) vs. infants of normal-weight mothers (*n* = 26)	Infant fecal samples at 1 and 6 months of age	FISH qPCR	Higher weights of mothers were correlated with higher concentrations of *Bacteroides*, *Clostridium*, and *Staphylococcus*, and lower concentrations of the *Bifidobacterium* group prevalence of *Akkermansia muciniphila*, *Staphylococcus*, and *Clostridium difficile* groups were lower in infants of normal-weight mothers
Galley et al. 2014 [126]	Children of obese (*n* = 26) vs. nonobese mothers	Fecal samples from children 18–27 months of age	16S ribosomal RNA (rRNA) sequencing)	Effects of maternal obesity on offspring’s gut microbiota were stronger among children of mothers of higher socioeconomic status Higher alpha and beta diversity in children of obese vs. nonobese mothers Children born to obese vs. nonobese mothers had greater abundances of *Parabacteroides* spp. and *Oscillibacter* spp., as well as lower *Blautia* spp. and *Eubacterium* spp.
Mueller et al, 2016 [128]	Neonates (*n* = 18) born vaginally (5 to overweight mothers), neonates (*n* = 56) by elective C-section (26 to overweight mothers)	Second-day fecal samples from neonates	16S ribosomal RNA (rRNA) sequencing	Compared to neonates delivered vaginally to normal-weight mothers, microbiota of neonates born to overweight or obese mothers were enriched in *Bacteroides* and depleted in *Enterococcus*, *Acinetobacter*, *Pseudomonas*, and *Hydrogenophilus*
Gestational Diabetes Mellitus				
Hu et al., 2013 [140]	Newborns (*n* = 23): 5 from mothers with DM, 5 from mothers with GDM, 13 from mothers with no diabetes	Meconium samples	16S ribosomal RNA (rRNA) sequencing	The phylum *Bacteroidota* and the genus *Parabacteriodes* were enriched in the meconium in the DM group compared to the nondiabetes group
Bassols et al., 2016 [44,155]	Placentas from women with GDM (*n* = 11) and from control women (*n* = 11)	Placenta	16S ribosomal RNA (rRNA) sequencing	*Pseudomonadales* and *Acinetobacter* showed lower relative abundance in women with GDM compared to control Increase in placental *Acinetobacter* ratio was associated with a more adverse metabolic and inflammatory phenotype
Wang et al., 2018 [139]	Pregnant women and their neonates with and without GDM	Oral, pharyngeal, meconium, and amniotic fluid samples	16S ribosomal RNA (rRNA) sequencing	In the amniotic fluid of the GDM group, a lower relative abundance of *Anoxybacillus* and a higher relative abundance of *Corynebacterium* were detected In the meconium of the GDM group, a lower relative abundance of *Corynebacterium* and a higher relative abundance of *Enterobacter* were detected Microbes varied by the same trend between the maternal and neonatal microbiota
Vegetarian Diet				
None				
Artificial Sweeteners				
Laforest-Lapointe et al., 2021 [155]	Infants (*n* = 100) selected based on maternal sweetener consumption during pregnancy (50 nonconsumers and 50 daily consumers)	Infant fecal samples at 3 and 12 months of age	16S ribosomal RNA (rRNA) sequencing	Maternal sweetener consumption did not differ between clusters reflecting the maturation of gut microbiota but was associated with community-level shifts in infant’s gut bacterial taxonomy structure and depletion of several *Bacteroides* sp. in a certain cluster Nine bacterial taxa from *Bacteroides* sp. were enriched or depleted at high levels of maternal sweetener consumption at 12 months of age. Daily maternal sweetener consumption is associated with higher infant weight and altered microbiota composition
Alcohol Consumption				
Wang et al., 2021 [166]	Pregnant women and their neonates with (*n* = 10) and without (*n* = 19) alcohol consumption	Fecal samples of newborns within 48 h	16S ribosomal RNA (rRNA) sequencing	A positive relationship showed between *Megamonas* and newborns with maternal alcohol consumption

## 5. Modulation of Maternal Gut Microbiota for Offspring’s Benefits

Given the proven impact of maternal microbiota on fetal health and development perinatally and postnatally, modulation of gestational dysbiosis could have prophylactic potential regarding noncommunicable diseases such as obesity, immunoinflammatory disorders, and neurocognitive complications. Since modification of the microbiota can be carried out easily, prenatal maternal oral pro- and/or prebiotic treatment could represent a safe, effective, and cheap interventional tool for disease prevention of the offspring.

### 5.1. Probiotics

Probiotics are live, beneficial microorganisms found in certain foods and supplements. They are thought to help to restore the physiological balance of the intestinal microbiota community. Most probiotic intervention studies are restricted to the use of *Lactobacilli* and *Bifidobacteria* strains. The beneficial effects of these strains are complex. They promote colonization resistance, limit mucosal adherence of pathogens, strengthen mucosal integrity, and enhance local immune defense, thereby reducing inflammation [170].

Most studies in the field of probiotics in pregnancy have focused on either the clinical outcome in pregnant women or in their offspring. Controversy exists regarding the preventive and useful effects of probiotics on the development of immune-mediated allergic disorders. While several clinical trials revealed the beneficial effect of maternal probiotics on lowering the risk of allergic conditions [171,172,173,174,175,176,177,178,179,180,181,182], others failed to confirm an advantage of probiotic treatment [183,184,185,186]. Regarding obesity, perinatal probiotic treatment could modify the growth pattern of the child by restricting excessive weight gain during the first years of life. Probiotic effects on GDM occurrence and symptoms have been intensively studied, with promising results [183,184,185,186,187,188,189,190].

Limited data are available on the mechanism of action and on the effect of probiotics on the maternal–fetal gut microbiota axis. There is good evidence that maternally derived probiotic bacteria can colonize the gastrointestinal tract of infants and persist there for 1–2 years [176,181,191,192,193,194,195,196]. However, another study revealed that the probiotic strain *Lactobacillus rhamnosus GG* increased the infant gut colonization by *Bifidobacterium* spp, but not by itself when administered to mothers in late pregnancy. This suggests that probiotics may promote fetal seeding with other bacteria, probably through the action of bacterial metabolites [197]. There is also the possibility that different probiotic bacteria could have different abilities to be transferred from the mother to the infant [173]. Furthermore, maternal dietary probiotic intake led to the modulated expression of TLR-related genes in the placenta and fetal intestinal tract, interfering thereby with fetal immune system development [175]. Since there is no consensus about the real impact of maternal probiotic intake on fetal gut microbiota composition and health issues, further investigations are needed [169].

### 5.2. Prebiotics

Prebiotics are food compounds that promote the growth and/or activity of beneficial microorganisms. The most common example is oligosaccharides resistant to digestion in the small intestine. Modulating maternal gut microbiota through the administration of prebiotics during pregnancy could be a safer alternative than probiotic consumption, as suggested by animal experiments [198]. In a mouse model of atopic dermatitis, prenatal maternal supplementation with a fructo-oligosaccharide modulated the intestinal microbiome of the offspring and suppressed the increase in clinical skin severity score and scratching behavior in offspring [199]. Prebiotic oligofructose treatment of diet-induced obese pregnant rats was found to reduce maternal energy uptake, reduce gestational weight gain, and prevent increased adiposity in dams and their offspring [122]. A high-fiber diet of mice led to marked suppression of allergic airways disease in the offspring’s, which could be mediated in utero via modulation of maternal gut microbiota [82]. Only few studies exist reporting offspring’s modulated gut microbiota and health benefits from maternal prebiotic intake in mice [198,199,200,201,202].

## 6. Conclusions

Studies of germ-free animals revealed that the absence of a healthy microbiota is associated with deficits in immune and neuronal development, impaired stress adaptation, and metabolic dysfunction later in life [83,86,203,204]. This observation was supported by plenty of human studies describing altered microbiota composition and dysbiosis as possible etiologic factors of several noncommunicable diseases in humans. Thus, the establishment and maintenance of a healthy microbiota are crucial for human health. Among the human microbiota at different body sites, the intestinal microbiota is thought to be the most important concerning health effects.

The main source of a newborn’s intestinal microbiota is the maternal gut. During the last decade, it has become obvious that maternal commensal microbes or their products are transferred to the fetus through the placenta in utero and/or postnatally. There they affect the composition of the fetal/neonatal intestinal microbial community. The establishment of a healthy early gut microbiota in life has long-lasting effects on the offspring’s metabolism and immune system and lowers the risk of developing a range of diseases later in life. Therefore, it is conceivable that any factors that affect the establishment of a healthy gut microbiota in the newborn/infant can potentially have a long-term impact on the offspring’s health. Maternal dietary factors could have a significant impact on the maternal–fetal microbiota axis, and modulation of dysbiotic gut microbiota may be beneficial both for the mother and also for her baby.

## 7. Future Directions

Despite convincing results discussed in this review, knowledge on some critical points and major events is still missing in this field. The possibility of an in utero translocation of maternal gut microbiota to the fetus should be further investigated, and phases of the process should be determined. Further investigations are needed to explore the complex association between early gut microbiota composition and its long-term effects on adult health issues. Determination of key bacteria or bacterial shifts in the background of certain noncommunicable diseases would be a major step forward.

Beneficial effects of prenatal pro- and prebiotic treatment on offspring’s health were also shown, although it requires detailed studies regarding the type, the dosage, and the timing of pro-/prebiotic intake during pregnancy. Furthermore, there are some investigational microbiome therapeutics, which may have preventive potential on the maternal transfer of dysbiotic microbiota to the fetus/newborn.

Healthy maternal diet has a significant impact on healthy maternal gut microbiota, which, in turn, affects the formation of the fetal/newborn intestinal microbiota. However, this is only the beginning. Maintenance of eubiosis is critical for long-lasting beneficial effects in terms of preventing noncommunicable diseases. As postnatal development of the child proceeds, the role of the mother’s bacteria becomes less important, and environmental factors occur. However, a good start in life ensured by the maternal gut microbiota remains always a major health determining factor.
